# Assessment of Primary Health Care for rural workers exposed to pesticides

**DOI:** 10.11606/s1518-8787.2020054001455

**Published:** 2020-01-14

**Authors:** Alessandra Cristina Pupin Silvério, Isarita Martins, Denismar Alves Nogueira, Marco Antônio Santos Mello, Edilaine Assunção Caetano de Loyola, Miriam Monteiro de Castro Graciano

**Affiliations:** I Universidade José do Rosário Vellano. Curso de Medicina. Alfenas - MG, Brasil; II Universidade Federal de Alfenas. Faculdade de Farmácia. Curso de pós-graduação em Ciências Farmacêuticas. Alfenas - MG, Brasil; III Universidade Federal de Alfenas. Instituto de Ciências Exatas. Programa de pós-graduação em Estatística Aplicada e Biometria. Alfenas - MG, Brasil; IV Universidade José do Rosário Vellano. Faculdade de Medicina. Alfenas - MG, Brasil; V Universidade Federal de Lavras. Departamento de Ciências da Saúde. Alfenas - MG, Brasil

**Keywords:** Rural Workers, Occupational Health, Agrochemicals, poisoning, Personal Protection Equipment, Working Conditions, Primary Health Care, Rural Health Services

## Abstract

**OBJECTIVE:**

To evaluate the attributes of Primary Health Care (PHC) for rural workers; to analyze sociodemographic conditions, history of poisoning and hospitalizations for pesticides and use of personal protective equipment; and to verify exposure to pesticides by determining bioindicators.

**METHODS:**

Cross-sectional, descriptive-analytical study with a sample of 1,027 rural workers living in municipalities belonging to a regional health department in Southern Minas Gerais, whose PHC is governed by the Family Health Strategy model. We used the adult version of the Primary Care Assessment Tool (PCATool Brazil) and a structured questionnaire to collect socioeconomic data, history of poisoning and hospitalization for pesticides and use of personal protective equipment. Blood samples were collected to measure biomarkers of pesticide exposure and signs of renal and hepatic sequelae.

**RESULTS:**

Low education was prevalent, as well as the intense contact of workers with pesticides. Frequent use of personal protective equipment was higher among men, as was the history of poisoning and hospitalizations for pesticides. Rates of 20% poisoning, 15% liver disease and 2% nephropathy were detected. Signs of hepatotoxicity were more frequent in men. Gender differences were all statistically significant. Regarding PHC, only the attribute “degree of affiliation” had a high score. None of the poisoning cases detected in the study were previously diagnosed.

**CONCLUSIONS:**

Despite the high coverage of the Family Health Strategy, occupational risk and its consequences have not been detected by health services, which do not seem oriented to primary care, even lacking their essential attributes. There is a need for immediate and effective adaptation of public policies regarding the health of rural workers, with adequate training of teams and review of the portfolio of PHC services offered.

## INTRODUCTION

The Brazilian Constitution of 1988 determines that it is the citizen’s right and the state’s duty to ensure the health needs of the population with quality services ^1^ . However, years after the institution of this universal right, equity and comprehensive care are still major challenges ^1 - 4^ .

In rural areas of Brazil, health services have lower supply and quality, as well as greater difficulty in accessing the Primary Health Units, resulting in an inequality between supply and demand in urban and rural areas ^5^ . In the latter, the demand for care is almost always motivated by acute diseases ^6^ . Thus, the population receives services based on demand and not on organized supply according to its supposed or perceived needs ^6 - 8^ .

In this context, the regional health department under study, located in the south of Minas Gerais, has 21.96% of its population residing in rural areas, much higher than the national average (15.65%) and the state average (14.7%) largely due to work in coffee farms ^9^ . Due to the mountainous relief, mild climate and favorable soil, the south of Minas Gerais is one of the largest coffee producers in the world ^10^ .

Consequently, pesticide use is high in this region, and rural workers are daily exposed to their harmful effects. In addition, chronic and acute poisonings have been reported, as in other regions of the country ^11^ . Between 2007 and 2014, 25,106 cases of pesticide poisoning from agricultural use were reported to the Ministry of Health, an average of 3,125 cases per year and eight daily poisonings in Brazil – and it is estimated that for each case of notified poisoning, 50 are not notified ^11^ .

The study by Silvério et al. ^12^ , which aimed to assess occupational exposure to pesticides in rural workers using genotoxicity testing, bioindicators and clinical evaluation, showed the health situation of rural workers from Southern Minas Gerais. The group exposed to pesticides showed alterations in both laboratory and clinical evaluations, especially damage to the central nervous system ^12^ .

Therefore, given the magnitude and relevance of the problem, it should be considered that the perception of occupational risks and preventive interventions, health promoters and educators are strengths of actions and services inherent to primary health care (PHC). This level of attention is the structuring element of health systems, showing four essential attributes (first contact access, integrality, longitudinality and care coordination) and three derived attributes (family orientation, community orientation and cultural competence) ^13^ . Thus, a primary care service can be considered really based on PHC when it has the four essential attributes, promoting the increase in its ability to interact with individuals and the community by also showing the derived attributes ^13^ .

Therefore, once the PHC is at the center of the health care network, its attributes must be evaluated, verifying the effectiveness of care on the population’s health. Thus, there is evidence of a growing association between better health outcomes and greater presence and extension of PHC attributes ^14^ .

Consequently, the objective was to evaluate the attributes of PHC in the health care offered to rural workers; to analyze sociodemographic conditions, history of poisoning and hospitalizations for pesticides and use of personal protective equipment (PPE); and to verify exposure to pesticides by determining bioindicators.

## METHODS

This is a descriptive-analytical cross-sectional study with a quantitative approach ^15^ , conducted in rural areas of a regional health department based in Alfenas, Minas Gerais, with 26 municipalities, whose PHC is primarily governed by the Family Health Strategy (FHS). According to the Brazilian Institute of Geography and Statistics (IBGE) ^9^ , the study’s target population was 66,266 working-age rural dwellers, of which 28,837 women between 18 and 60 years and 37,429 men between 18 and 65 years. Based on this population, the sample N was calculated.

The defined sample size was 1,038 respondents, with a 95% confidence and 3% margin of error. The stratification of the sample was made according to the number of municipalities belonging to the regional health department, totaling 26 sectors. The size in each sector was defined proportionally to that of the population living in rural areas of productive age per municipality.

The percentage participation of each sector in the sample composition and the number of interviews conducted in each one of them were also stratified by sex. The final study sample consisted of 1,027 research subjects, since in one of the sectors there was no cooperation for identification and selection. Data were collected in the rural FHS of each sector, after defining the geographic points to be visited, by the number of rural communities.

For data collection, two structured questionnaires were used, one for the survey of epidemiological and clinical data of rural workers, obtained from the health service of the Universidade de Campinas (Unicamp), and the Primary Care Assessment Tool (PCATool) ^14^ , prepared by Bárbara Starfield et al. ^13^ and validated in Brazil by Harzheim et al. ^16^ . Both instruments have structured questions that are easy to understand and simple to apply. Table 1 shows the essential and derived PHC attributes evaluated in PCATool. Information on biomarkers of pesticide poisoning was obtained by collecting 5.0 mL of blood sample in vacutainer tubes containing heparin and serum.

**Table 1:** Social and occupational risk characteristics of the rural population of the regional health department of Alfenas registered by rural teams of the Family Health Strategy. Minas Gerais, 2014–2015.

Variable	Men (n = 637)	Women (n = 390)	p
Age (years) ^a^	43.25 (13.50)	40.82 (12.85)	0.004 ^b^
Median	43	40	
Education (years) ^a^	5.62 (3.29)	6.01 (3.56)	0.265 ^b^
Median	4	4	
Contact time (years) ^a^	17.48 (10.23)	16.83 (10.67)	0.367 ^b^
Median	16	15	
Smoking (%)	39.04	21.09	< 0.001 ^c^
Drinking (%)	51.48	23.18	< 0.001 ^c^
Employment relationship (%)			< 0.001 ^c^
Owner	59.72	48.18	
Wage earner	22.40	22.40	
Sharecropper/tenant	11.82	6.25	
Other	6.07	22.92	
Worker’s function (%)			< 0.001 ^c^
Administrative	10.42	5.99	
Agricultural technician/agronomist	3.89	1.30	
Syrup applicator/preparer	13.22	2.86	
Family farming	72.47	89.84	
Pesticide contact time (%)			0.570 ^c^
3 – 5 Years	13.69	9.90	
6 – 10 years	18.20	8.85	
11 – 20 years	34.53	17.19	
> 20 years	27.84	16.93	
Pesticide application method (%)			< 0.001 ^c^
Coastal pump	75.58	41.41	
Hose	1.71	0.26	
Tractor without cabin	11.66	0.26	
Tractor with cabin	1.09	0.26	
Others	2.95	11.20	
Pesticide poisoning (%)	23.48	7.29	< 0.001 ^c^

^a^ Mean (standard deviation)

^b^ Mann-Whitney’s test

^c^ Chi-square Test

A pilot test was carried out with 50 rural workers from the municipality of Alfenas, in order to refine the tool, collect exposure data and train for blood sample collection. This allowed testing the research planning, answering questions about the application of the questionnaires and clarifying the subject for the interviewers.

Data were collected from June 2014 to June 2015, in which epidemiological and clinical variables such as sex, age, education, history of poisoning and/or hospitalization for pesticide poisoning and use of PPE were analyzed; occupational hazards, especially exposure and length of exposure to pesticides; pesticide exposure biomarkers established by plasma cholinesterase (PChE), erythrocyte cholinesterase (AChE) and total cholinesterase (TChE) activity; and sequelae signal biomarkers, evaluated by the dosage of aspartate aminotransferase (AST), alanine aminotransferase (ALT), gamma glutamyl transpeptidase (ƔGT) and serum creatinine. Finally, the attributes of PHC were evaluated through PCATool Brazil.

The adult version of the PCATool has 87 items. The initial three questions are not an attribute, they aim to identify which health unit the user has as reference and the degree of affiliation to this service, being scored from 1 to 4. The next questions are distributed between the essential attributes of PHC and the derivatives. The possible answers to the questions are: “definitely yes” (value 4), “probably yes” (value 3), “probably not” (value 2), “certainly not” (value 1) and “I don’t know/don’t remember” (value 9) ^14^ .

The scores for each of the attributes or their components are calculated by the simple arithmetic mean of the response values of their items. As a general result of the PCATool evaluation, there are two measures: the essential score, which is the average of the component scores of the essential attributes, and the general score, which is the previous value plus the scores of the derived attributes. These results characterize the degree of health service orientation towards PHC attributes ^14^ . All analyses of the scores were performed according to the guidelines of the PCAtool Brazil Manual ^14^ . The obtained scores were scaled as high (≥3) or low (<3), as proposed by Leão et al. ^17^ .

Regarding the evaluation of selected biomarkers, the analytical method used to determine pesticide exposure was the one proposed by Ellman et al. and modified by Harlin and Ross ^18^ . It is based on the colorimetric measurement of the rate of acetylthiocholine hydrolysis by blood cholinesterases.

For the interpretation of acetylcholine activity results, the regional reference value was used, since it was not possible to determine individual reference values for workers due to the prolonged exposure time without leave greater than 30 days. Regional reference values were estimated from the measurement of cholinesterase activity of 100 individuals of both sexes and without occupational exposure to pesticides residing in the Alfenas urban zone. The enzymatic activity range obtained for TChE was from 12.7 to 30.5%, for PChE from 1 to 6.4%, and for AChE from 31.1 to 59.4%.

For analysis of the other biomarkers (AST, ALT, γGT and serum creatinine), known to be altered by the use of pesticides ^19^ , we used Labtest^®^ commercial kits, with kinetic and enzymatic methodologies performed in biochemical automation equipment.

The data obtained were entered into a spreadsheet and later exported to a database of the SPSS version 17.0 program, from which the frequency analyses of categorical and descriptive variables of quantitative variables were performed. The chi-square test, Mann-Whitney test, and Fisher’s exact test were used with a 5% significance.

The study was registered in Plataforma Brasil and approved under opinion No. 149.718. All participants signed the Informed Consent Form.

## RESULTS

The sample showed that the rural working population in Southern Minas Gerais has low education and intense and prolonged contact with pesticides ( Table 1 ). Although direct contact with pesticides is equally common between men and women, men use PPE more frequently than women, who mostly do not use them at all. Despite this, the history of poisoning and hospitalizations for pesticide poisoning is more frequent in men than in women. These differences are all statistically significant ( Figure 1 ).

**Figure 1:** Exposure, protective measures and sequelae of pesticide management among rural working men and women in the regional health department of Alfenas, Minas Gerais.

Variable	Men	Women	P (Fisher’s exact test)
	
	%	%	
Direct contact with pesticides	99.2	98.2	0.016
History of prior poisoning	23.5	7.3	<0.001
History of hospitalization for contamination	66.4	4.4	0.029
Proper use of PPE	20.9	2.8	<0.001
Inappropriate use of PPE	60.8	34.3	<0.001
Failure to use PPE	18.4	62.9	<0.001

Changes in the dosage of all biomarkers of exposure or poisoning by pesticides were detected. In the study sample, 20% of the rural workers showed altered results in the total cholinesterase dosages and fractions. The hepatotoxicity index (15%) was high, more frequent in men than women, with statistically significant difference. There was no significant association with the degree of exposure, contact time, or alcohol consumption, which leads to infer its correlation with the chronicity of intoxication, more common in men ( Table 2 ).

**Table 2:** Biomarkers of the toxic action of pesticides in a rural population of the regional health department of Alfenas registered by rural teams of the Family Health Strategy. Minas Gerais, 2014–2015 (n = 1,000).

Biomarkers	Men	Women	p ^a^
	
	%	%	
Altered total cholinesterase	16.80	16.40	0.879
Altered erythrocyte cholinesterase	19.40	14.20	0.035
Altered plasma cholinesterase	3.60	2.10	0.180
Altered aspartate aminotransferase	13.90	4.30	<0.001
Altered alanine aminotransferase	13,20	4.30	<0.001
Altered glutamyl transpeptidase range	8.70	6.80	0.285
Altered serum creatinine	3.64	2.10	0.180

^a^ Chi-square Test

Observing the attributes of PHC, shown in Table 3 , only the degree of affiliation has a high score (≥3), as well as a statistically significant difference in the evaluation that men and women make of the item integrality: promotion and prevention actions.

**Table 3:** Mean scores of the attributes of primary health care provided to the rural population of the regional health department of Alfenas, Minas Gerais, obtained from PCATool Brazil.

Scores	Men			Women			p ^a^
	
	X̅	Mean	Standard deviation	X̅	Mean	Standard deviation	
Degree of affiliation	2.74	3.00	1.05	3.05	3.00	0.91	<0.001
First contact	2.76	2.83	0.52	2.76	2.83	0.48	0.686
Longitudinality	2.83	2.86	0.67	2.83	2.93	0.66	0.884
Care coordination	2.47	2.50	0.7	2.58	2.61	0.63	0.108
Integrality: available services	1.88	1.82	0.55	1.88	1.82	0.54	0.962
Integrality: promotion and prevention actions	2.03	2.00	0.72	1.97	1.92	0.70	<0.001
Family counseling	2.31	2.00	0.93	2.29	2.33	0.91	0.839
Community orientation	2.46	2.50	0.88	2.50	2.50	0.89	0.560
Essential	2.55	2.54	0.43	2.57	2.53	0.40	0.710 ^b^
Derived	2.38	2.42	0.74	2.40	2.33	0.75	0.985
General	2.52	2.50	0.45	2.53	2.51	0.43	0.806 ^b^

PCATool: Primary Care Assessment Tool

^a^ Chi-square Test

^b^ Mann-Whitney’s test

## DISCUSSION

Using PCATool as a PHC assessment tool is today one of the most important and reliable methods of analysis. A review by Prates et al. ^3^ found 155 published articles; however, none verified the PHC of rural residents exposed to pesticides, which adds real importance to this study.

Noting the association between the extension of PHC attributes and better health outcomes ^16^ , it can be stated that only the presence of health services not directed to primary care will not result in improvement in the living and health conditions of the population ^13^ . The analysis of the health situation of a rural population covered by the FHS showed here corroborates this hypothesis.

However, not only the health care model adopted directly influences the health levels of a population, but also its educational level. A cohort study with 18,825 respondents showed a negative correlation between educational level and biological risk factors ^20^ . Literature shows an association between education, degree of information and awareness of occupational risks ^21^ . Thus, the level of education is important for the correct use of PPE, for obtaining information on the risks of exposure to pesticides and for understanding the information on product labels, which can directly contribute to the levels of intoxication ^22^ . The rural workers of southern Minas Gerais have, for the most part, a schooling restricted to Elementary School I, which certainly influences the inappropriate use or even the complete rejection of the use of PPE.

An essential aspect for the care of workers by rural FHS refers to the domiciliation of work, observed in this research, that is, the performance of paid productive activities in the dwelling space and in the worker’s home area ^23^ . In such cases, work is commonly performed in makeshift environments, exposing workers and their families to health risks without any monitoring. Therefore, almost always only the FHS teams have access to these locations, making it possible to identify risk situations and health effects of this population and initiate an intervention ^23^ .

We found that 55.5% of those surveyed are owners of their workplaces and 74.3% work with family farming – i.e., they are mostly small farmers who develop their activities through direct employment of their own and family workforce. In this context, the use of PPE is often neglected, especially by women. Among their duties in family farming are activities for which they find it unnecessary to use PPE, such as washing clothes used to spray pesticides. Men often act in the storage and administration of pesticides, usually by costal pumps (80.3%) in the sample studied. Thus, women end up having exposure at lower concentrations, a fact that justifies the lower occurrence of chronic poisoning in this sex. On the other hand, men have a more direct contact and in higher concentrations with the pesticides, giving more common acute cases of intoxication ( Figure 1 ). Given the neglect of PPE use in 79% of men and 97% of women, it is not surprising that almost 20% of this population has changes in total cholinesterase dosages and fractions ( Table 2 ).

Cholinesterase activity determination is routine for the assessment of occupational and environmental exposure to anticholinesterases and recommended by the American Conference of Governmental Industrial Hygienists (ACGIH) ^24 - 27^ . The determination of PChE activity is considered a biological indicator of internal dose and AChE activity a biomarker of the effect of these pesticides.

The activity of these enzymes is used as a biomarker of environmental contamination and changes mainly in the face of pesticides. Exposure to them shows that these AChE inhibitions may be greater in chronic exposures with incomplete recovery after many exposures ^25 , 28^ . AChE inhibition is more sensitive than PChE in the case of chronic exposure, with cumulative inhibiting effects ^25^ . Literature indicates a significant relationship between exposure to these pesticides and AChE inhibition in rural populations and occupationally exposed workers, and this inhibition is considered a biomarker of neurotoxicity ^12 , 25^ .

However, the dosage of cholinesterases as an indicator has limitations, such as intra and interindividual variation and nonspecificity. Some factors, such as age, gender, race, nutritional status and pathologies, especially in the liver, may affect its activity ^24 , 25^ .

Despite these limitations, the determination of cholinesterase activity is a well-established method for health surveillance of workers exposed to pesticides. The truly significant difficulty in terms of public health is that the Brazilian health system finances only the dosage of PChE and not AChE. In fact, an AChE index of 19.4% in men and 14.2% in women, compared with a PChE index of 3.6% in men and 2.1% in women ( Table 2 ) shows the limitation of PChE to assess chronic pesticide exposure and the importance of including AChE dosing in the PHC service portfolio and procedures in Brazil.

In the 26 municipalities in the survey, rural family health teams were unaware of the evaluation by cholinesterase dosages as well as the clinical importance of monitoring rural workers chronically handling pesticides. In addition, the study population misjudged the integrality of care, especially regarding promotion and prevention actions, generating a median score of 1.82 ( Table 3 ). These data corroborate the observation in other regions of the country that municipal health services are not prepared to deal with cases of pesticide poisoning, lacking trained human resources for adequate technical advice and laboratory infrastructure for diagnosis and management of cases. Consequently, cases are underreported, making the real knowledge of pesticide poisoning in the country even more difficult ^22 , 29 , 30^ .

Thus, we infer that the rural worker population is vulnerable to exposure and pesticide poisoning due to the toxicity of these substances, low education level, inadequate use or even non-use of PPE ^30^ and unpreparedness of health services. The study of Bortolotto et al. ^29^ showed that living in rural areas is one of the most relevant aspects to negatively define the quality of life of a population.

It is noteworthy that all cases of poisoning detected in this study had their diagnoses established by the researchers. Access to the secondary level of care at the university’s occupational diseases outpatient clinic was the main benefit established in the research consent form.

Failure to detect a frequent health problem in a high-risk population that has access to primary care services can be explained by the lack of the essential attributes of primary care for this population. Therefore, health services that do not have the structural capacity with competence to perform anything more than spontaneous demand, incomplete regarding the range of services offered and the coordination of care, and without adequate complementation of other points of care, not considering family and community orientation and cultural competence, cannot be considered PHC strategies ^13^ .

Interestingly, the only attributes with a statistically significant difference in assessment between men and women were the degree of affiliation and integrality: promotion and prevention actions. This may be due to the fact that women, because they use services more than men, recognize a lower efficiency in health promotion and disease prevention actions offered to them.

Broadening the network of basic health services in the country, without adequate preparation of teams for action oriented to the attributes of primary care, even though it has had an impact on some health indicators in the past, is not being effective in reducing risks and damages to the health of rural workers. Besides the recommendation of adequate preparation of the rural FHS teams, the results of this study infer the importance of including the AChE dosage in the PHC service and procedures portfolio, since the enzymatic activity in the erythrocyte fraction more accurately reflects chronic exposure and cumulative for monitoring pesticide exposure and proper reporting, with consequent proper case management and health education of the population.

There is a need for immediate and effective adaptation of public policies regarding the health of rural workers. Autonomous farmers have as their only health resource the rural FHS, entities that should be prepared for health protection and promotion but are not even qualified for the effective diagnosis of cases, often being restricted to the identification of acute poisonings.

In other words, the absence of professional strategies that make workers aware of occupational health risks is serious. Thus, where individual and social vulnerability is most prominent, due to its high degree of affiliation with the FHS, the programmatic axis must intervene in a consistent harm reduction policy.

Thus, it is necessary to implement training programs for all FHS teams, aiming to properly serve the rural population. In addition, a set of measures involving regulation, health promotion activities and alternatives such as agroecology need to be discussed. The book *Dossiê ABRASCO: Um Alerta Sobre os Impactos dos Agrotóxicos* ^19^ , for example, along with complaints of the indiscriminate use of pesticides, denote the importance of the initiative of family and agroecological agriculture in the production of healthy food and in the construction of a more sustainable society. This dossier corroborates the statement that Brazil is moving in the opposite direction to several countries in which there has been a reduction in the use of pesticides, with a greater incentive to consume healthy, organic and agroecological foods, without reducing productivity and economic gains in food production.

**Chart 1:** Definition of PHC attributes and their evaluation by PCATool.

PHC Referral Service		
Attribute	Definition	Items evaluated in PCAtool
Degree of affiliation	How much the user identifies with the service.	A) Three items. This module defined the service to which the user was affiliated.
**Essential Attributes**		
First contact access	Access and use of health services whenever necessary.	B) First contact access (utilization): three items. The extent of access for each type of use (check-up consultation, follow-up, or if they wish to consult with the specialist); C) First contact access (accessibility): 12 items. Service structure, such as location and times.
Longitudinality	Understood as the professional-subject temporal relationship of attention, leading to the establishment of a strong mutual trust.	D) 14 items. Continuous attention over time.
Coordination	Understood as the integration of all care that the user receives and needs with other health services.	E) Coordination (integration of care): eight items. Synchronized articulation between various services and actions (reference/counter-reference); F) Coordination (information system): three items. Quality of records.
Integrality	Represented by actions of promotion, prevention, cure and rehabilitation appropriate to the context of PHC, recognizing the biopsychosocial character of the health-disease-illness process.	G) Integrality (services available): 22 items. Services considered basic present in the unit itself and resolution of the service; H) Integrality (services provided): 13 items for women and 11 items for men. Prevention and Health Promotion
**Derived attributes**		
Family counseling	Understood as the knowledge of family factors that interfere in the health-disease-illness process by the health team.	I) Three items. The recognition of family factors in the determination and treatment of the disease, that is, considering the family as the subject of attention.
Community orientation	Understood as recognition of community health needs.	J) Six items. Environmental and community factors in the determination and treatment of the disease, guiding the services for the benefit of the population.
Cultural competence	It means adapting health services to the cultural specificities of the community served.	Not included in adult PCATool version.

PHC: primary health care; PCATool: Primary Care Assessment Tool
