# Supplementary material for: Assessment of Primary Health Care for rural workers exposed to pesticides
Source: Rev Saude Publica. 2020 Jan 14;54:9. doi: 10.11606/s1518-8787.2020054001455 (PMC6961967; doi:10.11606/s1518-8787.2020054001455)
Supplement: Translation

# Translation: Avaliação da atenção primária à saúde de trabalhadores rurais expostos a praguicidas

**Authors:** Alessandra Cristina Pupin Silvério, Isarita Martins, Denismar Alves Nogueira, Marco Antônio Santos Mello, Edilaine Assunção Caetano de Loyola, Miriam Monteiro de Castro Graciano

**Affiliations:** I >Universidade José do Rosário Vellano. Curso de Medicina. Alfenas-MG, Brasil; II Universidade Federal de Alfenas. Faculdade de Farmácia. Curso de pós-graduação em Ciências Farmacêuticas. Alfenas-MG, Brasil; III Universidade Federal de Alfenas. Instituto de Ciências Exatas. Programa de pós-graduação em Estatística Aplicada e Biometria. Alfenas-MG, Brasil; IV Universidade José do Rosário Vellano. Faculdade de Medicina. Alfenas-MG, Brasil; V Universidade Federal de Lavras. Departamento de Ciências da Saúde. Alfenas-MG, Brasil

**Keywords:** Trabalhadores Rurais, Saúde do Trabalhador, Agroquímicos, envenenamento, Equipamento de Proteção Individual, Condições de Trabalho, Atenção Primária à Saúde, Serviços de Saúde Rural

## Abstract

**OBJETIVO:**

Avaliar os atributos da atenção primária à saúde (APS) na assistência à saúde de trabalhadores rurais; analisar condições sociodemográficas, histórico de intoxicação e internações por agrotóxicos e uso de equipamentos de proteção individual; e verificar a exposição aos praguicidas pela determinação de bioindicadores.

**MÉTODOS:**

Estudo transversal, descritivo-analítico, com amostra de 1.027 trabalhadores rurais residentes em municípios pertencentes a uma superintendência regional de saúde do sul de Minas Gerais, cuja APS é regida pelo modelo da Estratégia Saúde da Família. Utilizou-se o Instrumento de Avaliação da Atenção Primária (PCATool Brasil) versão adulto e um questionário estruturado para coleta de dados socioeconômicos, histórico de intoxicação e internação por agrotóxicos e uso de equipamentos de proteção individual. Foram coletadas amostras sanguíneas para dosagem de biomarcadores de exposição a praguicidas e de sinais de sequelas renais e hepáticas.

**RESULTADOS:**

A baixa escolaridade foi prevalente, bem como o contato intenso dos trabalhadores com praguicidas. O uso frequente de equipamentos de proteção individual foi maior entre os homens, assim como o histórico de intoxicação e de internações por agrotóxicos. Detectaram-se índices de 20% de intoxicação, 15% de hepatopatia e 2% de nefropatia. Os sinais de hepatotoxicidade foram mais frequentes em homens. As diferenças entre sexos foram todas estatisticamente significantes. Com relação à APS, apenas o atributo “grau de afiliação” apresentou escore elevado. Nenhum dos casos de intoxicação detectados no estudo tinha diagnóstico prévio.

**CONCLUSÕES:**

A despeito de uma alta cobertura da Estratégia Saúde da Família, o risco ocupacional e suas consequências não têm sido detectados pelos serviços de saúde, que se apresentam como não orientados à atenção primária, carecendo mesmo de seus atributos essenciais. Percebe-se a necessidade de adequação imediata e efetiva das políticas públicas no que concerne à saúde do trabalhador rural, com adequada capacitação das equipes e revisão da carteira de serviços da APS ofertados.

## INTRODUÇÃO

A Constituição Brasileira de 1988 determina que a saúde é direito do cidadão e dever do Estado para garantir o atendimento das necessidades de saúde da população com serviços de qualidade^1^. Pode-se observar que, anos após a instituição desse direito universal, a equidade e a integralidade do cuidado ainda são grandes desafios^1 - 4^.

As regiões rurais do Brasil apresentam quanto ao serviço de saúde menor oferta e qualidade, além de maior dificuldade de acesso as Unidade Básicas de Saúde, resultando em uma desigualdade entre oferta e procura nas áreas urbanas e rurais^5^. Nas últimas, a procura por atendimento é quase sempre motivada por doenças agudas^6^. Assim, são disponibilizados para a população serviços fundamentados na demanda e não na oferta organizada em função de suas necessidades supostas ou percebidas^6 - 8^.

Nesse contexto, a regional de saúde em estudo, localizada no sul de Minas Gerais, possui 21,96% de sua população residindo em zona rural, índice bem superior à média nacional (15,65%) e do estado (14,7%) devido, em grande parte, ao trabalho em lavouras de café^9^. Em função do relevo montanhoso, clima ameno e solo propício, a região sul-mineira é uma das maiores produtoras de café do mundo^10^.

Consequentemente, o uso de praguicidas é elevado nessa região, e os trabalhadores rurais estão cotidianamente expostos a seus efeitos nocivos. Ademais, intoxicações crônicas e agudas têm sido registradas, assim como em outras regiões do país^11^. Entre 2007 e 2014 foram notificados ao Ministério da Saúde 25.106 casos de intoxicação por praguicidas de uso agrícola, uma média de 3.125 casos por ano e de oito intoxicações diárias no Brasil – e para cada caso de intoxicação notificada, estima-se que outros 50 não o sejam^11^.

O trabalho de Silvério et al.^12^, que objetivou avaliar a exposição ocupacional a agrotóxicos em trabalhadores rurais utilizando teste de genotoxicidade, bioindicadores e avaliação clínica, demonstrou a situação de saúde de trabalhadores rurais do sul de Minas Gerais. O grupo exposto aos praguicidas apresentou alterações tanto nas avaliações laboratoriais quanto na clínica, com destaque para danos ao sistema nervoso central^12^.

Portanto, dada a magnitude e relevância do problema, deve-se levar em conta que a percepção de riscos ocupacionais e intervenções preventivas, promotoras da saúde e educadoras são pontos fortes das ações e serviços inerentes à atenção primária à saúde (APS). Esse nível de atenção é o elemento estruturante dos sistemas de saúde, apresentando quatro atributos essenciais (acesso de primeiro contato, integralidade, longitudinalidade e coordenação do cuidado) e três atributos derivados (orientação familiar, orientação comunitária e competência cultural)^13^. Logo, um serviço de atenção básica pode ser considerado realmente embasado na APS quando possuir os quatro atributos essenciais, promovendo o aumento da sua capacidade de interação com os indivíduos e com a comunidade ao apresentar também os atributos derivados^13^.

Portanto, estando a APS no centro da rede de atenção à saúde, seus atributos devem ser avaliados, permitindo verificar a eficácia da atenção sobre a saúde da população. Assim, são observadas evidências da crescente associação entre melhores desfechos de saúde e maior presença e extensão dos atributos da APS^14^.

Assim, o objetivo foi avaliar os atributos da APS na assistência à saúde ofertada a trabalhadores rurais; analisar condições sociodemográficas, histórico de intoxicação e de internações por agrotóxicos e uso de equipamentos de proteção individual (EPI); e verificar a exposição aos praguicidas por meio da determinação de bioindicadores.

## MÉTODOS

Trata-se de estudo descritivo-analítico, do tipo transversal, com abordagem quantitativa^15^, realizado em zonas rurais de uma superintendência regional de saúde com sede em Alfenas, Minas Gerais, contemplando 26 municípios, cuja APS é regida prioritariamente pelo modelo da Estratégia Saúde da Família (ESF). No ano de elaboração do projeto, a população-alvo do estudo, segundo o Instituto Brasileiro de Geografia e Estatística (IBGE)^9^, era constituída por 66.266 moradores rurais em idade produtiva, sendo 28.837 mulheres entre 18 e 60 anos e 37.429 homens entre 18 e 65 anos. Foi sobre ela que se calculou o N da amostra.

O tamanho amostral definido foi de 1.038 entrevistados, com uma confiança de 95% e 3% de margem de erro. A estratificação da amostra foi feita em função do número de municípios pertencentes à regional de saúde, totalizando 26 setores. O tamanho em cada setor foi definido de forma proporcional ao da população residente em zona rural em idade produtiva por município.

A porcentagem de participação de cada setor na composição da amostra e o número de entrevistas realizadas em cada um deles foram também estratificados por sexo. A amostragem final do estudo foi constituída por 1.027 sujeitos de pesquisa, pois em um dos setores não se obteve cooperação para a identificação e seleção. Os dados foram coletados nas ESF rurais de cada setor, após definição dos pontos geográficos a serem visitados, pelo número de comunidades rurais.

Para a coleta de dados utilizaram-se dois questionários estruturados, um para levantamento de dados epidemiológicos e clínicos dos trabalhadores rurais, obtido no serviço de saúde do trabalhador da Universidade Estadual de Campinas (Unicamp), e o Instrumento de Avaliação da Atenção Primária (PCATool)^14^, elaborado por Bárbara Starfield et al.^13^e validado no Brasil por Harzheim et al.^16^. Ambos os instrumentos possuem perguntas estruturadas de fácil compreensão e simples aplicação. Os atributos essenciais e derivados da APS avaliados no PCATool estão detalhados no Quadro 1 . Já as informações sobre biomarcadores da intoxicação por praguicidas foram obtidas por meio da coleta de 5,0 mL de amostra sanguínea em tubos *vacutainer* contendo heparina e para soro.

**Quadro 1:** Definição dos atributos da APS e sua avaliação pelo PCATool.

Serviço de referência da APS		
Atributo	Definição	Itens avaliados no PCAtool
Grau de afiliação	O quanto o usuário se identifica com o serviço.	A) Três itens. Este módulo definia o serviço ao qual o usuário era afiliado.
**Atributos essenciais**		
Acesso de primeiro contato	Acesso e uso do serviço de saúde sempre que necessário.	B) Acesso de primeiro contato (utilização): três itens. A extensão do acesso para cada tipo de uso (consulta de check-up, retorno ou se desejo de passar com o especialista); C) Acesso de primeiro contato (acessibilidade): 12 itens. Estrutura do serviço, como localização e horários.
Longitudinalidade	Compreendida como a relação temporal profissional-sujeito da atenção, levando ao estabelecimento uma forte confiança mútua.	D) 14 itens. Atenção contínua ao longo do tempo.
Coordenação	Entendida como a integração de todo o cuidado que o usuário recebe e precisa com os outros serviços de saúde.	E) Coordenação (integração dos cuidados: oito itens. Articulação sincronizada entre diversos serviços e ações (referência/contrarreferência); F) Coordenação (sistema de informações): três itens. Qualidade dos registros.
Integralidade	Representada por ações de promoção, prevenção, cura e reabilitação adequadas ao contexto da APS, reconhecendo o caráter biopsicossocial do processo saúde-doença-adoecimento.	G) Integralidade (serviços disponíveis): 22 itens. Serviços considerados básicos presentes na própria unidade e resolutividade do serviço; H) Integralidade (serviços prestados): 13 itens para as mulheres e 11 itens para os homens. Prevenção e promoção da saúde.
**Atributos derivados**		
Orientação familiar	Compreende o conhecimento dos fatores familiares que interferem no processo saúde-doença-adoecimento pela equipe de saúde.	I) Três itens. O reconhecimento de fatores familiares na determinação e tratamento da doença, ou seja, considerando a família como sujeito da atenção.
Orientação comunitária	Entendida como o reconhecimento das necessidades de saúde da comunidade.	J) Seis itens. Os fatores ambientais e comunitários na determinação e tratamento da doença, orientando os serviços para o benefício da população.
Competência cultural	Significa adaptar os serviços de saúde às especificidades culturais da comunidade atendida.	Não contemplada na versão PCATool adulto.

APS: atenção primária à saúde; PCATool: Instrumento de Avaliação da Atenção Primária

Foi realizado o teste-piloto com 50 trabalhadores rurais do município de Alfenas, a fim de realizar refinamentos no instrumento, coleta dos dados de exposição e treinamento para a coleta de amostras sanguíneas. Isso permitiu testar o planejamento da pesquisa, sanar dúvidas quanto à aplicação dos questionários e esclarecer o tema para os entrevistadores.

Os dados foram coletados no período de junho de 2014 a junho de 2015, no qual foram analisadas variáveis epidemiológicas e clínicas tais como: sexo, idade, escolaridade, histórico de intoxicação e/ou internação por intoxicação com praguicidas e uso de EPI; riscos ocupacionais, principalmente exposição e tempo de exposição a praguicidas; biomarcadores de exposição à praguicida, estabelecidos pela atividade da colinesterase plasmática (BChP), colinesterase eritrocitária (AChE) e colinesterase total (ChT); e biomarcadores de sinais de sequelas, avaliados pela dosagem da aspartato aminotransferase (AST), da alanina aminotransferase (ALT), gama glutamil transpeptidase (γGT) e creatinina sérica. Por fim, avaliaram-se os atributos da APS por meio do PCATool Brasil.

O PCATool versão adulto possui 87 itens. As três perguntas iniciais não constituem um atributo, visam identificar qual unidade de saúde o usuário tem como referência e o grau de afiliação a esse serviço, sendo pontuado de 1 a 4. As próximas perguntas são distribuídas entre os atributos essenciais da APS e os derivados. As respostas possíveis as indagações são: “com certeza sim” (valor 4), “provavelmente sim” (valor 3), “provavelmente não” (valor 2), “com certeza não” (valor 1) e “não sei/não lembro” (valor 9)^14^.

Os escores para cada um dos atributos ou seus componentes são calculados pela média aritmética simples dos valores das respostas de seus itens. Como resultado geral da avaliação pelo PCATool, tem-se duas medidas: o escore essencial, que é a média dos escores dos componentes dos atributos essenciais, e o escore geral, que é o valor anterior acrescido dos escores dos atributos derivados. Esses resultados caracterizam o grau de orientação do serviço de saúde para os atributos da APS^14^. Todas as análises dos escores foram realizadas segundo as orientações do Manual do PCAtool Brasil^14^. Os escores obtidos foram escalonados como alto (≥ 3) ou baixo (< 3), conforme proposto por Leão et al.^17^.

Com relação à avaliação dos biomarcadores selecionados, o método analítico utilizado para determinar a exposição a praguicidas foi o proposto por Ellman et al. e modificado por Harlin e Ross^18^. Ele baseia-se na medida colorimétrica da velocidade de hidrólise da acetiltiocolina pelas colinesterases sanguíneas.

Para interpretação dos resultados de atividade da acetilcolina, foi utilizado o valor de referência regional, já que não foi possível determinar valores de referência individual para os trabalhadores devido ao tempo prolongado de exposição sem afastamento superior a 30 dias. Os valores de referência regional foram estimados a partir da medida da atividade das colinesterases de cem indivíduos de ambos os sexos e sem exposição ocupacional a praguicidas residentes na zona urbana de Alfenas. O intervalo de atividade enzimática obtido para a ChT foi de 12,7 a 30,5%, para a BChP foi de 1 a 6,4%, e para a AChE foi de 31,1 a 59,4%.

Para análise dos demais biomarcadores (AST, ALT, γGT e creatinina sérica), reconhecidamente alterados pelos usos de praguicidas^19^, utilizaram-se kits comercias da marca Labtest^®^, com as metodologias cinéticas e enzimáticas realizadas em equipamento de automação bioquímica.

Os dados obtidos foram digitados em planilha eletrônica e posteriormente exportados para um banco de dados do programa SPSS versão 17.0, a partir do qual se realizaram as análises de frequências das variáveis categóricas e descritiva das variáveis quantitativas. Foram utilizados os testes do qui-quadrado, Mann-Whitney, t e exato de Fisher, considerando 5% de significância.

O estudo foi inscrito na Plataforma Brasil e aprovado sob o parecer nº 149.718. Todos os participantes assinaram o termo de consentimento livre e esclarecido.

## RESULTADOS

A amostra evidenciou que a população trabalhadora da zona rural no sul de Minas Gerais possui baixa escolaridade e contato intenso e prolongado com praguicidas ( Tabela 1 ). Embora o contato direto com praguicidas seja igualmente frequente entre homens e mulheres, os homens utilizam mais frequentemente os EPI de forma adequada do que as mulheres, que em sua maioria não os utilizam de forma alguma. A despeito disso, a história de intoxicação e internações por intoxicação com praguicidas é mais frequente em homens do que nas mulheres. Essas diferenças são todas estatisticamente significativas ( Figura 1 ).

**Tabela 1:** Características sociais e de risco ocupacional da população de zona rural da regional de saúde de Alfenas cadastradas por equipes rurais da Estratégia Saúde da Família. Minas Gerais, 2014–2015.

Variáveis	Homens (n = 637)	Mulheres (n = 390)	p
Idade (anos)^a^	43,25 (13,50)	40,82 (12,85)	0,004^b^
Mediana	43	40	
Escolaridade (anos)^a^	5,62 (3,29)	6,01 (3,56)	0,265^b^
Mediana	4	4	
Tempo de contato (anos)^a^	17,48 (10,23)	16,83 (10,67)	0,367^b^
Mediana	16	15	
Tabagismo (%)	39,04	21,09	< 0,001^c^
Etilismo (%)	51,48	23,18	< 0,001^c^
Relação de trabalho (%)			< 0,001^c^
Proprietário	59,72	48,18	
Assalariado	22,40	22,40	
Meeiro/arrendatário	11,82	6,25	
Outro	6,07	22,92	
Função do trabalhador (%)			< 0,001^c^
Administrativa	10,42	5,99	
Técnico agrícola/agrônomo	3,89	1,30	
Aplicador/preparador de calda	13,22	2,86	
Agricultura familiar	72,47	89,84	
Tempo de contato com praguicidas (%)			0,570^c^
3–5 anos	13,69	9,90	
6–10 anos	18,20	8,85	
11–20 anos	34,53	17,19	
> 20 anos	27,84	16,93	
Modo de aplicação dos praguicidas (%)			< 0,001^c^
Bomba costal	75,58	41,41	
Mangueira	1,71	0,26	
Trator sem cabine	11,66	0,26	
Trator com cabine	1,09	0,26	
Outros	2,95	11,20	
Intoxicação por praguicidas (%)	23,48	7,29	< 0,001^c^

^a^Média (desvio-padrão)

^b^Teste de Mann-Whitney

^c^Teste do qui-quadrado

**Figura 1:** Exposição, medidas protetoras e sequelas do manejo de praguicidas entre homens e mulheres trabalhadores de zona rural na regional de saúde de Alfenas, Minas Gerais.

Variáveis	Homens	Mulheres	P (Teste exato de Fisher)
	
	%	%	
Contato direto com praguicida	99,2	98,2	0,016
História de intoxicação prévia	23,5	7,3	< 0,001
História de internação por contaminação	66,4	4,4	0,029
Utilização adequada de EPI	20,9	2,8	< 0,001
Utilização de EPI de forma inadequada	60,8	34,3	< 0,001
Não utilização de EPI	18,4	62,9	< 0,001

Foram detectadas alterações na dosagem de todos os biomarcadores de exposição ou intoxicação por praguicidas avaliados. Na amostra em estudo, 20% dos trabalhadores rurais apresentaram resultados alterados nas dosagens de colinesterase total e frações. Elevado foi o índice de hepatotoxicidade (15%), mais frequente em homens do que mulheres, com diferença estatisticamente significativa. Não houve associação significativa ao grau de exposição, tempo de contato, nem ao consumo de álcool, o que leva a inferir a sua correlação à cronicidade da intoxicação, mais comum em homens ( Tabela 2 ).

**Tabela 2:** Biomarcadores da ação tóxica de praguicidas em população da zona rural da regional de saúde de Alfenas cadastrados por equipes rurais da Estratégia Saúde da Família. Minas Gerais, 2014–2015 (n = 1.000).

Biomarcadores	Homens	Mulheres	p^a^
	
	%	%	
Colinesterase total alterada	16,80	16,40	0,879
Colinesterase eritrocitária alterada	19,40	14,20	0,035
Colinesterase plasmática alterada	3,60	2,10	0,180
Aspartato aminotransferase alterada	13,90	4,30	< 0,001
Alanina aminotransferase alterada	13,20	4,30	< 0,001
Gama glutamil transpeptidase alterada	8,70	6,80	0,285
Creatinina sérica alterada	3,64	2,10	0,180

^a^Teste do qui-quadrado

Observando os atributos da APS, mostrados na Tabela 3 , apenas o grau de afiliação apresenta escore elevado (≥ 3), bem como uma diferença estatisticamente significativa na avaliação que homens e mulheres fazem do ítem integralidade: ações de promoção e prevenção.

**Tabela 3:** Média dos escores dos atributos da atenção primária à saúde dos serviços prestados à população de zona rural da regional de saúde de Alfenas, Minas Gerais, obtidos por meio do PCATool Brasil.

Escores	Homens			Mulheres			p^a^
	X̅	Média	Desvio-padrão	X̅	Média	Desvio-padrão	
Grau de afiliação	2,74	3,00	1,05	3,05	3,00	0,91	< 0,001
Primeiro contato	2,76	2,83	0,52	2,76	2,83	0,48	0,686
Longitudinalidade	2,83	2,86	0,67	2,83	2,93	0,66	0,884
Coordenação do cuidado	2,47	2,50	0,7	2,58	2,61	0,63	0,108
Integralidade: serviços disponíveis	1,88	1,82	0,55	1,88	1,82	0,54	0,962
Integralidade: ações de promoção e prevenção	2,03	2,00	0,72	1,97	1,92	0,70	< 0,001
Orientação familiar	2,31	2,00	0,93	2,29	2,33	0,91	0,839
Orientação comunitária	2,46	2,50	0,88	2,50	2,50	0,89	0,560
Essencial	2,55	2,54	0,43	2,57	2,53	0,40	0,710^b^
Derivado	2,38	2,42	0,74	2,40	2,33	0,75	0,985
Geral	2,52	2,50	0,45	2,53	2,51	0,43	0,806^b^

PCATool: Instrumento de Avaliação da Atenção Primária

^a^Teste do qui-quadrado

^b^Teste de Mann-Whitney

## DISCUSSÃO

O uso do PCATool como instrumento de avaliação da APS é hoje um dos mais importantes e confiáveis métodos de análise. Uma revisão feita por Prates et al.^3^encontrou 155 artigos publicados; no entanto, nenhum verificou a APS de moradores de zona rural expostos a agrotóxicos, o que agrega real importância a este estudo.

Observando a associação entre a extensão dos atributos da APS e melhores desfechos de saúde^16^, pode ser afirmado que somente a presença de serviços de saúde não direcionados à atenção primária não resultará em melhoria nas condições de vida e saúde da população^13^. A análise da situação de saúde de uma população rural coberta pela ESF aqui apresentada corrobora essa hipótese.

Entretanto, não só o modelo assistencial de saúde adotado influencia diretamente os níveis de saúde de uma população, mas também o seu grau de escolaridade. Um estudo de coorte com 18.825 pesquisados evidenciou correlação negativa entre grau de escolaridade e fatores de risco biológico^20^. A literatura demonstra associação entre escolaridade, grau de informação e conscientização dos riscos ocupacionais^21^. Assim, o grau de escolaridade é importante para o uso correto de EPI, para a obtenção de informação sobre os riscos de exposição a praguicidas e para o entendimento das informações presentes nos rótulos dos produtos, o que pode contribuir diretamente para os níveis de intoxicação^22^. Os trabalhadores rurais do sul mineiro têm, em sua grande maioria, uma escolaridade restrita ao Ensino Fundamental I, o que certamente influencia o uso inadequado ou mesmo a completa rejeição do uso de EPI.

Um aspecto essencial para o cuidado dos trabalhadores pelas ESF rurais refere-se à domiciliação do trabalho, observada nesta pesquisa, ou seja, a realização de atividades produtivas remuneradas no espaço de moradia e no peridomicílio do trabalhador^23^. Nesses casos, o trabalho é comumente desenvolvido em ambientes improvisados, expondo os trabalhadores e suas famílias a riscos para a saúde, sem qualquer monitoramento. Portanto, quase sempre apenas as equipes da ESF têm acesso a esses locais, possibilitando identificar situações de risco e efeitos sobre a saúde dessa população e iniciar uma intervenção^23^.

Verificou-se que 55,5% dos pesquisados são proprietários de seus locais de trabalho e 74,3% trabalham com agricultura familiar – ou seja, são, em sua maioria, pequenos produtores rurais que desenvolvem suas atividades mediante emprego direto da força de trabalho própria e da família. Nesse contexto, frequentemente o uso do EPI é negligenciado, principalmente pelas mulheres. Entre suas atribuições na agricultura familiar, há atividades para as quais elas julgam desnecessário o uso do EPI, como lavagem de roupas usadas na pulverização dos praguicidas. Já o homem muitas vezes atua na guarda e administração dos agrotóxicos, geralmente por meio de bombas costais (80,3%) na amostra estudada. Assim, a mulher acaba tendo uma exposição em concentrações mais baixas, fato que justifica a menor ocorrência de intoxicações crônicas nesse sexo. Por outro lado, o homem tem um contato mais direto e em concentrações mais altas com os praguicidas, conferindo quadros de intoxicação aguda mais comuns ( Figura 1 ). Dado o descaso com o uso de EPI em 79% dos homens e 97% das mulheres, não é nada surpreendente o fato de que quase 20% desta população apresenta alterações nas dosagens de colinesterase total e frações ( Tabela 2 ).

A determinação da atividade das colinesterases é rotineira para avaliação da exposição ocupacional e ambiental aos anticolinesterásicos e recomendada pela *American Conference of Governmental Industrial Hygienists* (ACGIH)^24 - 27^. A determinação da atividade da BChP é considerada um indicador biológico de dose interna e a atividade da AChE um biomarcador de efeito desses agrotóxicos.

A atividade dessas enzimas é utilizada como um biomarcador de contaminação ambiental e se altera principalmente diante de agrotóxicos. A exposição a eles mostra que essas inibições das AChE podem ser maiores em exposições crônicas com recuperação incompleta após muitas exposições^25 , 28^. A inibição da AChE é mais sensível do que a BChP no caso da exposição crônica, sendo os efeitos cumulativos inibitórios^25^. A literatura aponta relação significativa entre a exposição a esses agrotóxicos e a inibição das AChE em populações rurais e de trabalhadores expostos ocupacionalmente, e por isso essa inibição é considerada um biomarcador de neurotoxicidade^12 , 25^.

Entretanto, a dosagem das colinesterases como indicador apresenta limitações, como variação intra e interindividual e inespecificidade. Alguns fatores, tais como idade, gênero, raça, estado nutricional e patologias, principalmente hepáticas, podem afetar sua atividade^24 , 25^.

Apesar destas limitações, a determinação da atividade das colinesterases é método consagrado para vigilância em saúde do trabalhador exposto a praguicidas. A dificuldade verdadeiramente significativa em termos de saúde pública é o fato de o sistema de saúde brasileiro financiar apenas a dosagem da BChP e não da AChE. De fato, um índice de AChE de 19,4% nos homens e 14,2% em mulheres, em comparação com um índice de BChP de 3,6% em homens e 2,1% em mulheres ( Tabela 2 ) demonstra a limitação da BChP para avaliar a exposição crônica aos praguicidas e a importância da inclusão da dosagem da AChE na carteira de serviços e procedimentos de APS no Brasil.

Nos 26 municípios da pesquisa, as equipes de saúde da família de zona rural desconheciam a avaliação por dosagens de colinesterases, bem como a importância clínica do monitoramento de trabalhadores rurais que manuseiam cronicamente praguicidas. Agregando-se a isso, a população em estudo avaliou mal a integralidade do cuidado, especialmente no quesito ações de promoção e prevenção, gerando uma mediana de escore igual a 1,82 ( Tabela 3 ). Esse dado corrobora a observação, em outras regiões do país, de que os serviços municipais de saúde não estão preparados para lidar com casos de intoxicação por praguicidas, carecendo de recursos humanos treinados para aconselhamento técnico adequado e infraestrutura laboratorial para diagnóstico e manejo dos casos. Consequentemente, há subnotificação de casos, dificultando ainda mais o conhecimento real da intoxicação por agrotóxicos no país^22 , 29 , 30^.

Pode-se assim inferir que a população de trabalhadores rurais encontra-se vulnerável à exposição e às intoxicações por praguicidas, devido ao grau de toxicidade dessas substâncias, à baixa escolaridade, uso inadequado ou mesmo não uso de EPI^30^e despreparo dos serviços de saúde. O trabalho de Bortolotto et al.^29^demonstrou que residir em zona rural é um dos aspectos mais relevantes para definir negativamente a qualidade de vida de uma população.

É importante observar que todos os casos de intoxicação detectados neste estudo tiveram seus diagnósticos estabelecidos pelos pesquisadores. O acesso ao nível secundário de atenção no ambulatório de doenças ocupacionais da universidade foi o principal benefício estabelecido no termo de consentimento da pesquisa.

A não detecção de um problema de saúde frequente em uma população de alto risco e que tem acesso a serviços de atenção primária pode ser explicada por essas não possuirem os atributos essenciais da atenção primária para essa população. Portanto, os serviços de saúde que não apresentam capacidade estrutural com competência para realizar algo mais que a demanda espontânea, incompletos no que diz respeito à amplitude de serviços oferecidos e à coordenação da atenção e sem complementação adequada dos outros pontos de atenção, não considerando a orientação familiar e comunitária e a competência cultural, não podem ser considerados estratégias da APS^13^.

É interessante que os únicos atributos com diferença estatisticamente significativa de avaliação entre homens e mulheres tenham sido o grau de afiliação e a integralidade: ações de promoção e prevenção. Isso pode ser devido pelo fato de que as mulheres, por usarem mais os serviços do que os homens, reconhecem uma menor eficiência nas ações de promoção da saúde e prevenção de doenças a elas ofertadas.

Ampliar a rede de serviços básicos de saúde no país, sem adequado preparo das equipes para uma ação orientada aos atributos da atenção primária, ainda que tenha produzido impacto em alguns indicadores de saúde no passado, não está sendo efetivo para a redução de riscos e danos à saúde de trabalhadores rurais. Além da recomendação de adequado preparo das equipes de ESF rural, os resultados deste trabalho inferem a importância da inclusão da dosagem da AChE na carteira de serviços e procedimentos da APS, visto que a atividade enzimática na fração eritrocitária reflete de forma mais fidedigna a exposição crônica e cumulativa para o monitoramento de exposição aos praguicidas e adequada notificação, com consequente manejo correto dos casos e educação em saúde da população adstrita.

Percebe-se a necessidade de uma adequação imediata e efetiva das políticas públicas no que concerne à saúde do trabalhador rural. Os agricultores autônomos têm como único recurso de saúde as ESF rurais, entidades que deveriam estar preparadas para a proteção e a promoção à saúde, mas que não estão capacitadas nem para o diagnóstico efetivo dos casos, restringindo-se muitas vezes à identificação de intoxicações agudas.

Ou seja, é grave a ausência de estratégias dos profissionais que sensibilizem os trabalhadores para os riscos ocupacionais associados à saúde. Assim, nos locais em que a vulnerabilidade individual e social se faz mais proeminente, por seu alto grau de afiliação com a ESF, é que o eixo programático deve intervir em uma política consistente de redução de danos.

Desse modo, é necessária a implantação de programas de capacitação para todas as equipes da ESF, visando atender de forma adequada a população rural. Além disso, um conjunto de medidas envolvendo regulação, atividades de promoção da saúde e alternativas como a agroecologia precisa ser discutido. O livro *Dossiê ABRASCO* : Um Alerta Sobre os Impactos dos Agrotóxicos^19^, por exemplo, junto com as denúncias do uso indiscriminado de praguicidas, denotam a importância da iniciativa da agricultura familiar e agroecológica na produção de alimentos saudáveis e na construção de uma sociedade mais sustentável. Esse dossiê vem corroborar com a afirmativa que o Brasil caminha na direção contrária a diversos países nos quais tem havido uma redução no uso dos agrotóxicos, com maior incentivo ao consumo de alimentos saudáveis, orgânicos e agroecológicos, sem, com isso, diminuir a produtividade e os ganhos econômicos na produção de alimentos.
